# Journey to kidney transplantation: patient dynamics, suspensions, transplantation and deaths in the Australian kidney transplant waitlist

**DOI:** 10.1093/ndt/gfad253

**Published:** 2023-11-28

**Authors:** Nicole L De La Mata, Victor Khou, James A Hedley, Patrick J Kelly, Rachael L Morton, Kate Wyburn, Angela C Webster

**Affiliations:** Sydney School of Public Health, Faculty of Medicine and Health, University of Sydney, Camperdown, NSW, Australia; Sydney School of Public Health, Faculty of Medicine and Health, University of Sydney, Camperdown, NSW, Australia; Sydney School of Public Health, Faculty of Medicine and Health, University of Sydney, Camperdown, NSW, Australia; Sydney School of Public Health, Faculty of Medicine and Health, University of Sydney, Camperdown, NSW, Australia; NHMRC Clinical Trials Centre, Faculty of Medicine and Health, University of Sydney, Camperdown, NSW, Australia; Sydney Medical School, Faculty of Medicine and Health, University of Sydney, Camperdown, NSW, Australia; Renal Department, Royal Prince Alfred Hospital, Camperdown, NSW, Australia; Sydney School of Public Health, Faculty of Medicine and Health, University of Sydney, Camperdown, NSW, Australia; NHMRC Clinical Trials Centre, Faculty of Medicine and Health, University of Sydney, Camperdown, NSW, Australia; Centre for Renal and Transplant Research, Westmead Hospital, Sydney, NSW, Australia

**Keywords:** Australia, kidney waitlist, outcomes, relisting, suspension

## Abstract

**Background:**

People on the kidney waitlist are less informed about potential suspensions. Disparities may exist among those who are suspended and who return to the waitlist. We evaluated the patient journey after entering the waitlist, including suspensions and outcomes, and factors associated with these transitions.

**Methods:**

We included all incident patients waitlisted for their first transplant from deceased donors in Australia from 2006 to 2019. We described all clinical transitions after entering the waitlist. We predicted the restricted mean survival time (unadjusted and adjusted) until first transplant by the number of prior suspensions. We evaluated factors associated with transitions using flexible survival models and clinical endpoints using Cox models.

**Results:**

Of 8466 patients waitlisted and followed over 45 757.4 person-years (median 4.8 years), 6741 (80%) were transplanted, 381 (5%) died waiting and 1344 (16%) were still waiting. A total of 3127 (37%) people were suspended at least once. Predicted mean time from waitlist to transplant was 3.0 years [95% confidence interval (CI) 2.8–3.2] when suspended versus 1.9 years (95% CI 1.8–1.9) when never suspended. Prior suspension increased the likelihood of further suspensions 4.2-fold (95% CI 3.8–4.6) and returning to the waitlist by 50% (95% CI 36–65) but decreased the likelihood of transplantation by 29% (95% CI 62–82). Death risk while waiting was increased 12-fold (95% CI 8.0–18.3) when currently suspended. Australian non-Indigenous males were 13% [hazard ratio (HR) 1.13 (95% CI 1.04–1.23)] and Asian males 23% [HR 1.23 (95% CI 1.06–1.42)] more likely to return to the waitlist compared with females of the same ethnicity.

**Conclusion:**

The waitlist journey was not straightforward. Suspension was common, impacted the chance of transplantation and meant waiting an average of 1 year longer until transplant. We have provided estimates for and factors associated with suspension, relisting and outcomes after waitlisting to support more informed discussions. This evidence is critical to further understand drivers of inequitable access to transplantation.

## Graphical Abstract

**Graphical Abstract:**
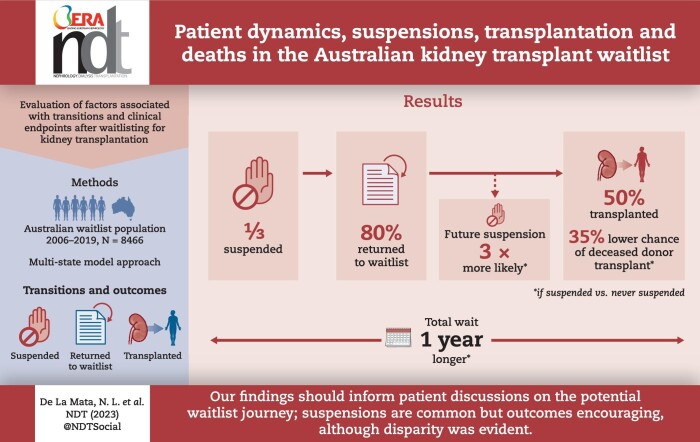

KEY LEARNING POINTS **What was known:**

- Certain groups are known to have disadvantaged access to the kidney waitlist, relating to both biological reasons and social determinants of health.
- A patient's journey on the waitlist is complex and may include suspensions and returning to the waitlist once or multiple times.
- The few studies reporting suspensions on the kidney waitlist are limited. They do not fully reflect the transient nature of suspensions and returning to the waitlist, nor have any studies used contemporary data to examine disparities relating to patient and clinical factors.

**This study adds:**

- Suspensions after entering the Australian kidney waitlist were common, occurring in approximately one-third of patients, and prior suspensions had a compounding effect, making it four times more likely to be suspended again and 50% more likely to return to the waitlist after suspension.
- Intersectional disadvantage was evident; only non-Indigenous males were more likely to return to the waitlist after suspension compared with their female counterparts.
- Prior suspension reduced the likelihood of deceased donor transplantation by 30% and led to a 1-year longer wait when forecasting over a 5-year period.

**Potential impact:**

- Suspensions offer an opportunity to improve health service delivery and support Indigenous people and females to return to the waitlist soon, or to list pre-emptively, to provide more possibilities for transplantation.
- Our work provides new evidence to better inform patients and support discussions on the expected journey on the kidney waitlist in Australia. Patients may find it helpful to know suspensions are common and most return to the waitlist and eventually receive a transplant.
- Our estimates of suspension, returning to the waitlist and death while waiting are informative for further modelling work, such as economic health models and predictive models for algorithm changes.

## INTRODUCTION

Kidney transplantation improves quality of life and overall survival for people with kidney failure, making it a compelling option for most [1]. Kidney transplantation is also more cost effective than any type of dialysis [2]. Hence, kidney transplantation is the preferred treatment for kidney failure from both an individual patient and a public health perspective. As demand for donor organs exceeds supply, access to transplantation from a deceased donor is via a waiting list. Australia has a universal public healthcare system that covers all expenses associated with dialysis and the transplantation process, including for living donors. Private dialysis providers also exist in Australia, but transplantation processes for deceased donors operate within large tertiary public hospitals. Eligibility criteria for placement on the kidney waitlist are informed by international clinical practice guidelines, with some national and regional variability [3]. Generally, criteria include a clinical indication of current or imminent kidney failure and a high likelihood of survival while waiting for a transplant and post-transplantation [4]. People may be excluded from the kidney waitlist due to multimorbidity that could significantly impact their life expectancy, such that they are unlikely to experience a net benefit from transplantation. Currently in Australia, people must have commenced dialysis, have a high likelihood of survival and benefit from transplantation to be eligible for waitlisting (prior to 2018, an estimated ≥80% 5-year post-transplant survival) [5]. There are no mandatory quality scores for waitlisting in Australian dialysis centres, nor is the reason for non-waitlisting or exclusion recorded in any administrative health register. Other potential exclusion criteria include intercurrent illness such as severe cardiovascular disease, uncontrolled infection and active or recent malignancy and an inability to comply with the complex medical regimen, which may be due to behavioural risk factors (e.g. ongoing drug misuse).

**Figure 1:**
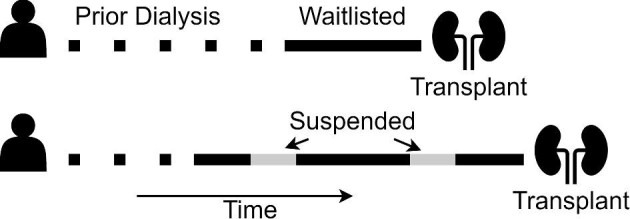
Example of how waiting times are calculated for allocation of kidney transplant after entering the Australian kidney waitlist.

Allocation algorithms are used worldwide to rank people on waitlists in order of preference whenever a donor kidney becomes available. While early allocation algorithms were largely based on waiting time, these have evolved to strike a balance between utility (the best use of organs for longevity) and equity (fair access to transplantation) [6]. Current allocation algorithms consider not only waiting time, but also factors such as age, human leukocyte antigen (HLA) matching, blood group and immunological reactivity between a potential recipient and donor. Waiting time calculations vary among countries and can begin from entry to the waitlist, initial referral for consideration or from dialysis commencement, even though patients may not be placed on the waitlist until sometime later. Expected waiting times also vary between and within countries. In Australia, these vary across states/territories due to differences in national and state allocations and differences in populations. Where waiting times are calculated from dialysis commencement (e.g. Australia and the USA), patients referred late may have more equitable access to transplantation. However, this leads to immortal time bias as patients cannot change their prior time spent on dialysis once they enter the waitlist (Fig. 1). People who spent more prior time on dialysis will likely have shorter waiting times once listed, as they are ranked as a higher priority and may also be less likely to be suspended from the waitlist. Conversely, people who spend less prior time on dialysis will likely spend more time active on the waitlist once listed.

Patients who have significantly longer waiting times will likely have a different waitlisting journey compared with others, which might include periods of temporary or permanent suspension from the waitlist. Temporary or permanent suspension from the waitlist may occur due to intercurrent illness (such as pneumonia or acute myocardial infarction), surgery or, more rarely, issues with social or mental health, or identification of a living donor, negating the need to wait for a deceased donor. In Australia, patients are counselled on expected waiting times for transplant and made aware that their prior dialysis time will contribute to their waiting time, but they are not directly involved in waiting list management and may not know their waitlist status in real time. They are less informed about how their waitlisting journey might unfold. Specifically, clinicians do not typically discuss in depth the process and likelihood of suspension episodes, how long they may be suspended for and the potential for never receiving a transplant, either due to death while waiting or permanent removal from the waitlist.

Prior waitlist studies have mainly focused on the transition from dialysis to waitlist and from waitlist to transplantation [7–10], with few studies examining suspensions on the waitlist [11–13]. Certain groups are known to have disadvantaged access to the waitlist or wait longer until transplant. These sometimes relate to biological reasons, such as HLA sensitisation or blood group, but may also relate to social determinants of health [14–17]. Women and ethnic minorities are known to have poorer access to waitlisting and transplantation [14, 15]. It is less clear how these factors may also impact suspensions and return to the waitlist. One US study evaluated the effect of a policy change to include suspended time into total waiting time on the subsequent return to the waitlist [11]. Other studies describe permanent suspensions [12] and ever suspended in the first year [13]. These had similar findings; African Americans and females were less likely to return to the waitlist [11], and African Americans and men were more likely to be permanently suspended [12]. However, these studies are limited, as they do not fully reflect the patient journey on the waitlist, including the transient nature of suspensions and returning to the waitlist, nor have any studies used contemporary patient cohorts. Disparities may exist in clinical outcomes between patients who are suspended and take longer to return to the waitlist and patients who are never suspended.

Our study objective was to describe the patient journey after first entering the waitlist, regardless of whether they receive a kidney transplant or not, including transitions between active waitlist and suspension, and factors associated with transitions on the kidney waitlist, with the ultimate aim of identifying the potential for reducing inequity.

## MATERIALS AND METHODS

### Study design and setting

We performed a population-based cohort study of all people who were waitlisted for a deceased-donor kidney transplant in Australia using data from the Australian and New Zealand Dialysis and Transplant Registry (ANZDATA). ANZDATA is a bi-national register of all people receiving kidney replacement therapy (dialysis or transplant) in Australia and New Zealand since 1965. Patient data include demographics, comorbidities and clinical data relating to their kidney disease. A more detailed description of ANZDATA is available elsewhere [18]. Patient waitlisting information, including date of activation and suspension (i.e. being made inactive) on the kidney waitlist since mid-2006, has become available in ANZDATA from data supplied by OrganMatch, the national system for waitlisting, organ matching and allocation in Australia.

### Participants

Our study population included all people who were ever waitlisted for their first kidney transplant in Australia between 1 July 2006 and 31 December 2019. These data were prior to the coronavirus disease 2019 pandemic in Australia, so there was no significant disruption to transplantation services that would impact our study. We excluded people who were already active on the waitlist (i.e. prevalent cases), who were pre-emptively transplanted, who were listed for their second or higher-order transplant or multi-organ transplants (i.e. kidney and other solid organ transplant).

This study received ethics approval from the University of Sydney (Project No. 2020/828).

### Outcomes

**Figure 2:**
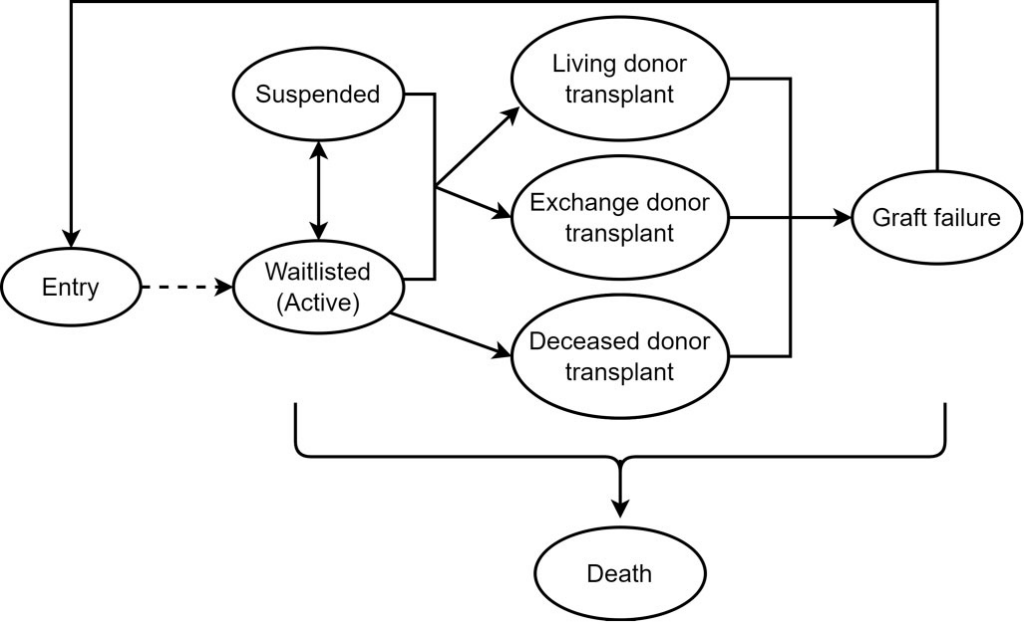
Schematic of clinical states and possible transitions between clinical states, indicated by arrows, used in our multistate waitlist model.

We considered all clinical states after entering the kidney waitlist as outcomes of interest. Clinical states included active on the waitlist; suspended (i.e. inactive); kidney transplant from deceased donor, transplanted from living donor or paired kidney exchange donor; graft failure after kidney transplant; and death at any time. Possible transitions are shown in Fig. 2. After entering the waitlist (active), patients could be suspended (inactive) at any time and return to waitlist (active). We considered all suspensions, regardless of duration or potential reason, to reflect the reality of the health system and patient journey on the waitlist. Deceased donor transplants could only occur from the waitlist (active). Transplants from living donors or paired kidney exchange donors could occur from either the waitlist (active) or while suspended (inactive). Transplant failure could only occur after transplantation. Patients could die or be censored from any clinical state due to no further follow-up (i.e. end of study period) or loss to follow-up.

We measured the time at risk from the date entering the waitlist to two endpoints: until the first transplant or death before transplant and until death or the end of follow-up. Patients were censored at 31 December 2019 or the date of their last follow-up if lost to follow-up. If graft failure and death occurred on the same date, patients transitioned immediately to the death state.

### Statistical analyses

We used two modelling approaches: a flexible parametric multistate model for specific transitions with recurrent events and accounting for competing risks and Cox proportional hazards models for clinical endpoints that ignored intermediate transitions. Further details are provided in the Supplementary Methods.

We used a flexible parametric multistate model to evaluate factors associated with two transitions after entering the waitlist: from waitlist to suspension and from suspension to waitlist. This was implemented using the flexsurv package in R (R Foundation for Statistical Computing, Vienna, Austria) [19]. We reported estimates of the cause-specific hazard ratios (HRs) for each transition. We used Cox proportional hazards models to evaluate factors associated with two clinical endpoints after entry to the waitlist: first transplant and death before the first transplant. We reported estimates of the cause-specific HRs for each clinical endpoint.

We fitted univariable models for each covariate and multivariable models including all covariates. Previous suspensions were fitted as a time-varying covariate and indicated the number of times a person had been suspended and returned to the waitlist. Covariates or factors of interest, selected *a priori*, included sex, age at waitlist entry (years), year of initial dialysis, ethnicity, blood group, comorbidity count, cause of kidney failure and Australian state/territory. Comorbidity count was the sum of any of the following comorbidities reported at dialysis initiation or since: cerebrovascular disease, coronary artery disease, peripheral artery disease, chronic lung disease and history of cancer. Australian state/territory was de-identified to avoid direct comparison of outcomes. An interaction term for sex and ethnicity was examined.

We predicted the restricted mean survival time (unadjusted and adjusted) until first transplant from a deceased donor by the number of prior suspensions with time horizons of 2 and 5 years using the strmst package in Stata (StataCorp, College Station, TX, USA) [20]. Prior suspension was included as a time-dependent variable. Covariates used for adjusted estimates included sex, age at waitlist entry (years), calendar year started dialysis, blood group and Australian state/territory.

Patient characteristics were summarised using absolute counts and proportions, overall and stratified by total number of times suspended. We also summarised the median time from first waitlisting to each transition until first transplant. We summarised the annual percentages in each clinical state until death or the end of follow-up (i.e. including clinical states after first transplant), annually up to 5 years of follow-up. We visualised these using Sankey plots, where each horizontal line represents an individual and depicts his/her flow between each of the clinical states.

Data were analysed using Stata 16 [21] and R version 4.0.3 [22].

## RESULTS

### Study characteristics

A total of 10 183 people were waitlisted for their first kidney transplant in Australia between 1 July 2006 and 31 December 2019. Of these, we excluded 1259 (12%) who were already active on the waitlist (prevalent cases) and 458 (4%) who had multi-organ transplants (predominantly simultaneous kidney–pancreas). The remaining 8466 (83%) were included in our study population. As expected, the distribution of time since waitlisting was different from the time since dialysis for each blood group (Supplementary Fig. S1). Overall, the median time from dialysis to deceased donor transplant was 2.6 years [interquartile range (IQR) 1.4–4.2], median time from entry to the waitlist to deceased donor transplant was 8.5 months (IQR 2.4–24.0) and median time active on the waitlist until deceased donor transplant (i.e. not including time suspended) was 7.3 months (IQR 2.2–19.7).

Among 8446 people waitlisted, most were never suspended (63%) while waiting for their first transplant (Table 1). However, 2112 (25%) were suspended once and 1015 (12%) were suspended twice or more. The patient characteristics were similar between these three mutually exclusive groups.

**Table 1:** Clinical characteristics of people who entered the waitlist for their first kidney transplant in Australia, 2006–2019, and by sex.

	Females		Males		Total	
Characteristics	*n*	%	*n*	%	*n*	%
Total	3098	37	5368	63	8466	100
Suspensions						
Never suspended	1932	62	3407	63	5339	63
Suspended once	759	25	1353	25	2112	25
Suspended twice or more	407	13	608	11	1015	12
Months per suspension, median (IQR)	2.7 (0.9–9.8)		3.2 (1.1–10.4)		3.0 (1.0–10.2)	
Total months suspended, median (IQR)	5.8 (1.9–20.7)		6.0 (1.9–20.2)		5.9 (1.9–20.4)	
Outcome						
Still waiting	335	11	509	9	844	10
Still suspended	196	6	304	6	500	6
Died while waiting	136	4	245	5	381	5
Transplanted	2431	78	4310	80	6741	80
Prior dialysis time (months), median (IQR)	12.2 (5.3–25.3)		11.7 (5.5–24.2)		11.9 (5.4–24.5)	
Age at waitlist entry (years)						
≤29	312	10	526	10	838	10
30–49	1077	35	1643	31	2720	32
50–64	1327	43	2395	45	3722	44
≥65	382	12	804	15	1186	14
Median (IQR)	51.7 (40.8–60.4)		53.5 (42.7–61.5)		52.8 (42.1–61.2)	
Year of kidney replacement therapy						
≤2007	590	19	983	18	1573	19
2008–11	841	27	1287	24	2128	25
2012–15	883	29	1656	31	2539	30
2016–19	784	25	1442	27	2226	26
State						
New South Wales	909	29	1608	30	2517	30
Victoria	852	28	1567	29	2419	29
Queensland	605	20	1005	19	1610	19
Western Australia	264	9	463	9	727	9
South Australia	259	8	449	8	708	8
Northern Territory	77	2	93	2	170	2
Tasmania	73	2	83	2	156	2
Australian Capital Territory	59	2	100	2	159	2
Ethnicity^a^						
Australian and New Zealander	1860	60	3418	64	5278	62
European	74	2	179	3	253	3
Aboriginal and Torres Strait Islander	209	7	268	5	477	6
Māori and Pacific Islander	134	4	187	3	321	4
Asian^b^	547	18	801	15	1348	16
Other^c^	103	3	236	4	339	4
Unknown	171	6	279	5	450	5
Blood group						
A	1140	37	2005	37	3145	37
AB^d^	118	4	241	4	359	4
B	419	14	713	13	1132	13
O	1421	46	2409	45	3830	45
Body mass index (kg/m^2^)						
Underweight (≤18.4)	202	7	166	3	368	4
Normal (18.5–24.9)	1147	38	1667	31	2814	34
Overweight (25.0–29.9)	829	27	1902	36	2731	33
Obese (≥30)	867	28	1559	29	2426	29
Not collected	53	–	74	–	127	–
Comorbidities at kidney replacement therapy						
Cerebrovascular disease	175	6	340	6	515	6
Coronary artery disease	431	14	1330	25	1761	21
Peripheral artery disease	269	9	709	13	978	12
Chronic lung disease	261	8	487	9	748	9
History of cancer	300	10	511	10	811	10
Diabetes						
Type 1	83	3	142	3	225	3
Type 2	606	20	1479	28	2085	25
Comorbidity count						
0	1735	56	2526	47	4261	50
1	859	28	1499	28	2358	28
2	320	10	751	14	1071	13
≥3	184	6	592	11	776	9
Smoking status						
Current	229	7	585	11	814	10
Former	777	25	2129	40	2906	34
Never	2092	68	2654	49	4746	56
Cause of kidney failure						
Diabetes	474	15	1166	22	1640	19
Hypertension/renal artery disease	203	7	530	10	733	9
Glomerulonephritis/immunoglobulin A nephropathy	1060	34	1906	36	2966	35
Polycystic kidney disease	561	18	667	12	1228	15
Other^e^	800	26	1099	20	1899	22

^a^Categorised based on the Australian Standard Classification of Cultural and Ethnic Groups 2016.

^b^Includes Northeast Asian (e.g. Chinese, Japanese, Korean, Taiwanese and Mongolian), Southeast Asian (e.g. Burmese, Cambodian, Filipino, Indonesian, Lao, Malay, Singaporean, Thailander, Timorese and Vietnamese) and South Asian (e.g. Indian, Nepalese, Sri Lankan and Pakistani).

^c^Includes African, Middle East, North American and South American.

^d^Includes one person with A2B.

^e^Most common other causes of kidney failure included congenital abnormalities of the kidney and urinary tract (31%), uncertain diagnosis (25%), drug and heavy metal toxicity (11%), obstructions (6%) and cancer (2%).

By the end of follow-up, 6741 (80%) people had received their first transplant (6136 deceased donor, 506 living donor, 99 living donor through paired kidney exchange program), 381 (5%) people died before transplantation (31 active on the waitlist, 350 while suspended) and 1344 (16%) were still waiting for a transplant (844 active on the waitlist,; 500 while suspended). Of the 6741 transplant recipients, 777 died after their first transplant, 615 had a graft failure and 121 had a second kidney transplant (112 deceased donor, 6 living donor, 3 paired kidney exchange donor). Of the 121 second transplant recipients, 7 died after their second transplant and 17 had a graft failure. There were no third or subsequent kidney transplants during our study follow-up.

### Suspension episodes until first transplant

The 8466 people waitlisted were followed for a total of 14 231.7 person-years observation time (median follow-up 10.8 months) from entry to the waitlist until their first kidney transplant, death before transplantation or last known follow-up/end of study period. The rates of suspension and death before transplant are presented in Supplementary Table S1.

**Figure 3:**
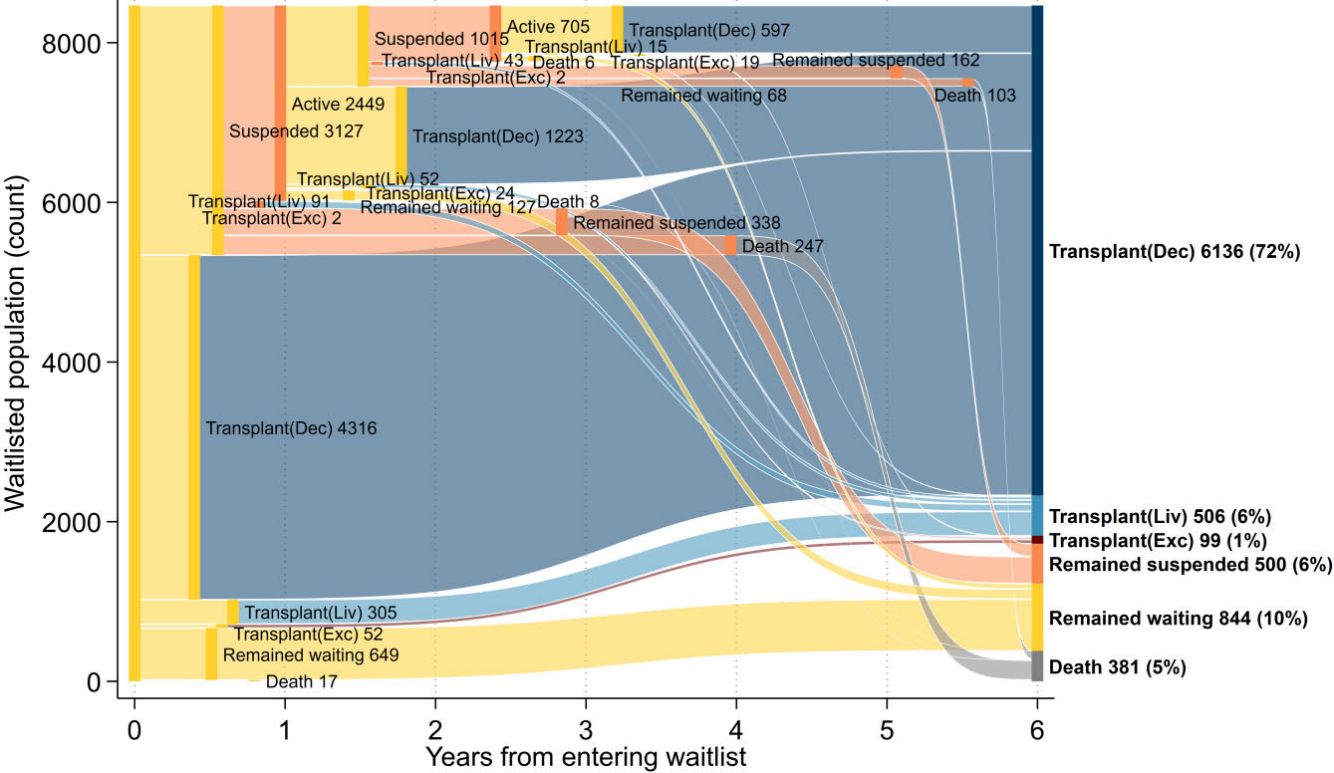
Waitlist transitions to first transplant at median transition time for people entering the waitlist for deceased donor transplant in Australia, 2006–2019. Active waitlist (i.e. waiting) is given in yellow, suspended in orange, deceased donor transplant (i.e. Transplant (Dec)) in dark blue, living donor transplant (i.e. Transplant (Liv)) in light blue, paired kidney exchange donor transplant (i.e. Transplant (Exc)) in red and death in grey.

For the 3127 people who were suspended at least once, the median time until their first suspension was 6.6 months (Fig. 3). After their first suspension, most (78%) returned to the waitlist and, of these, 53% eventually received a transplant. A smaller proportion did not return to the waitlist during follow-up (11%) and 8% died while suspended. A second or subsequent suspension occurred for 1015 people at a median time of 1.5 years after waitlist entry. Outcomes from subsequent suspensions were similar to those from the first suspension: 81% returned to the waitlist (79% eventually received a transplant), 7% died while suspended and 10% never returned to the waitlist. During each suspension, nearly half (48%) spent <3 months suspended, 17% spent 3–6 months suspended, 12% spent 6–12 months suspended and 24% spent ≥1 year suspended. The total suspension time per person was <6 months for 50%, 6 months–2 years for 27% and ≥2 years for 23% in our study cohort.

**Figure 4:**
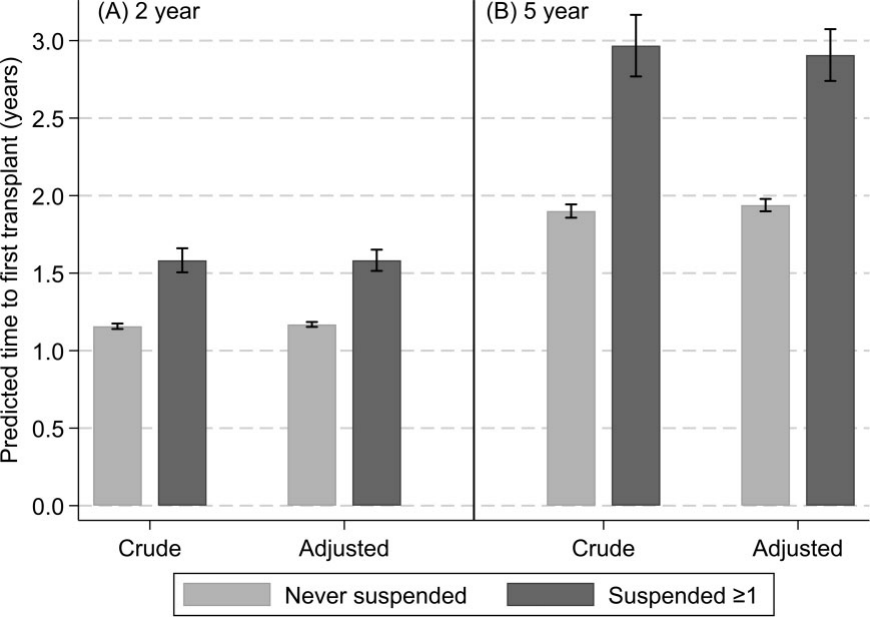
Predicted time from waitlist entry until first deceased donor transplant, not including prior dialysis time, for those never suspended and those suspended at least once, with time horizon of **(A)** 2 years and **(B)** 5 years. Adjusted for sex, age, year of dialysis initiation, blood group and Australian state/territory.

If you predict the patient will be transplanted within 2 years (i.e. time horizon of 2 years), the predicted time from waitlist entry until first deceased donor transplant, not including prior dialysis time, for those never suspended was 1.16 years [95% confidence interval (CI) 1.14–1.17] and 1.58 years (95% CI 1.51–1.66) for those suspended at least once. This mean difference was 0.43 years (95% CI 0.35–0.51; *P* < .001). If transplantation was expected to occur within 5 years (i.e. time horizon of 5 years), the predicted time to first transplant was 1.90 years (95% CI 1.86–1.94) for those never suspended and 2.97 years (95% CI 2.77–3.17) for those suspended at least once. This mean difference was 1.07 years (95% CI 0.86–1.27; *P* < .001). These predicted times to first transplant were similar when adjusting for additional patient factors (Fig. 4).

### Impact of waiting list suspensions and other factors on transitions to first transplant

**Figure 5:**
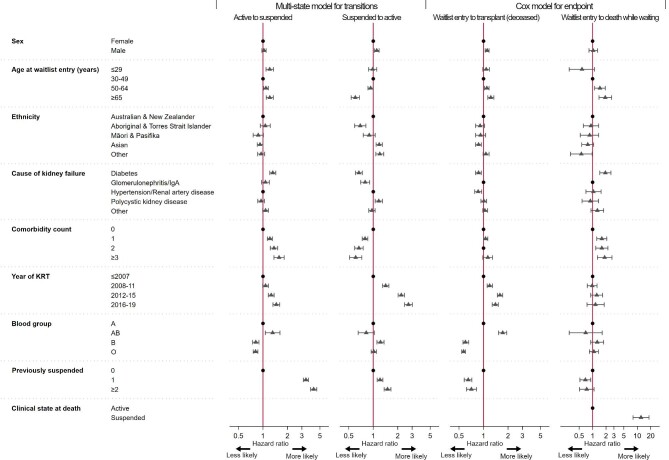
Factors associated with transitioning from active to suspended and suspended to active from the multivariable flexible parametric multistate model and with clinical endpoints from waitlist entry to transplant (deceased donor) and waitlist entry to death before transplant from the multivariable cause-specific Cox model. Estimates are adjusted for Australian state/territory. Refer to Supplementary Tables S3 and S6 for *P*-values.

Having been suspended from the waitlist increased the risk of further suspension and returning to the waitlist compared with no suspensions or suspension without returning to the waitlist (*P* < .001). The risk of another suspension was increased by 3.4 times [HR 3.37 (95% CI 3.13–3.62)] if only one prior suspension occurred and by 4.2 times [HR 4.18 (95% CI 3.83–4.57)] if two or more prior suspensions occurred (Fig. 5). Similarly, the likelihood of returning to the waitlist again was increased by 21% [HR 1.21 (95% CI 1.12–1.31)] after having one prior suspension and by 50% [HR 1.50 (95% CI 1.36–1.65)] after having two or more prior suspensions. Univariate results are presented in Supplementary Table S2.

Sociodemographic factors associated with an increased likelihood of being suspended and returning to the waitlist are presented in Fig. 5, with multivariate results presented in Supplementary Table S3. Of note, Aboriginal and Torres Strait Islanders were 31% [HR 0.69 (95% CI 0.59–0.81)] less likely to return to the waitlist after suspension compared with non-Indigenous Australian and New Zealanders. Sex and ethnicity were significant modifiers for the likelihood of returning to the waitlist (*P* < .001; Supplementary Table S4), but not for being suspended (*P* > .05). Males of Australian or New Zealand ethnicity (non-Indigenous) were 13% [HR 1.13 (95% CI 1.04–1.23), *P* < .01] and males of Asian ethnicity were 23% [HR 1.23 (95% CI 1.06–1.42), *P* < .01] more likely to return to the waitlist after suspension compared with females of the same ethnicity. However, males of Aboriginal, Torres Strait Islander, Māori or Pacific Islander ethnicity did not have this same advantage and were equally as (un)likely as their female counterparts to return to the waitlist after suspension (*P* = .08). There were also regional differences (*P* < .001) where patients from some Australian states/territories were more or less likely to be suspended or return to the waitlist compared with the reference group (Supplementary Table S3).

### Impact of waiting list suspensions and other factors on clinical endpoints after entering the waitlist

Having been suspended from the waitlist decreased chances of receiving a deceased donor transplant (*P* < .001). One prior suspension decreased the likelihood of transplant by 35% [HR 0.65 (95% CI 0.58–0.72)] and by 29% [HR 0.71 (95% CI 0.62–0.82)] with two or more prior suspensions (Fig. 5).

Dying without transplantation was strongly associated with being currently suspended (*P* < .001) and having prior suspensions (*P* = .017). Being currently suspended increased the risk of death before transplant 12-fold [HR 12.12 (95% CI 8.04–18.30)]. Prior suspension reduced the risk of death before transplant by 31% [HR 0.69 (95% CI 0.52–0.91)], likely due to survivorship bias, as only those well enough to return to the waitlist at least once could have a prior suspension. This relationship did not persist with two or more prior suspensions (*P* = .12).

Univariate and multivariate results for all factors associated with receiving a deceased donor kidney transplant and death before transplant after entering the waitlist from the Cox model are presented in Supplementary Tables S5 and S6. Sex and ethnicity were not modifiers of either receiving a deceased donor kidney transplant or death before transplant (*P* > .05). There were regional differences where in many Australian states/territories patients were more likely to receive a deceased donor transplant, and a few Australian states/territories where it was less likely for their patients to die while waiting compared with the reference group (Supplementary Table S6).

### Probabilities of waitlist transitions over time from first waitlisted

**Figure 6:**
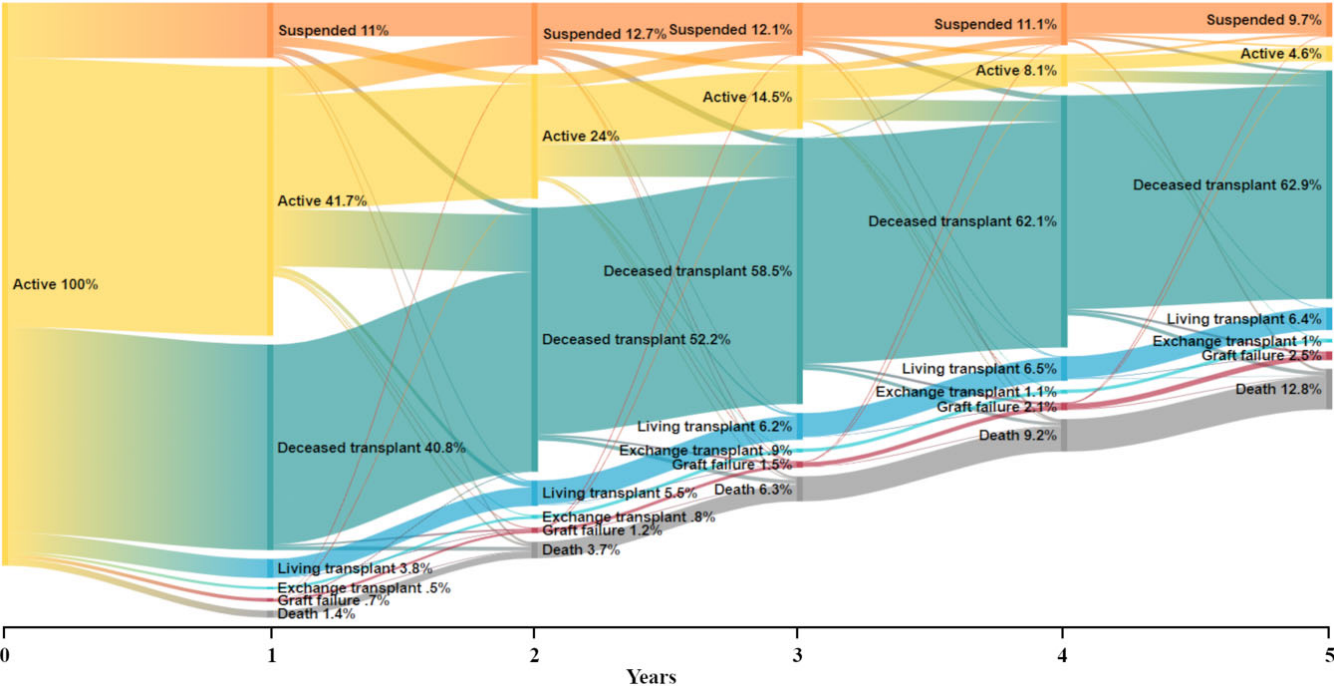
Sankey plot of all transitions once entering the waitlist for a deceased donor transplant and annual percentages in each clinical state in Australia, 2006–2019.

Considering patients’ entire journey after entering the kidney waitlist, through to first transplant and beyond, our study population was followed for a total of 45 757.4 person-years (median follow-up 4.8 years). The annual probability of being in each clinical state after entering the kidney waitlist are presented in Fig. 6.

At 1 year, 41.7% (95% CI 40.5–42.8) of patients were active on the waitlist and 11.0% (95% CI 10.3–11.7) were suspended. The percentage active on the waitlist approximately halved each year, reaching 4.6% (95% CI 4.0–5.2) at 5 years since first entering the waitlist. Conversely, suspensions remained relatively stable, reaching 12.7% (95% CI 11.9–13.5) at 2 years and 9.7% (95% CI 8.9–10.6) at 5 years.

After 1 year, 40.8% (95% CI 39.7–42.0) had a currently functioning deceased donor transplant and this steadily increased to nearly two-thirds of people at 5 years [62.9% (95% CI 61.5–64.3)]. Living donor transplants to those on the deceased donor waitlist were 3.8% (95% CI 3.4–4.3) in the first year and 6.4% (95% CI 5.7–7.1) by 5 years. Paired exchange donor transplants represented the minority of all transplants, at 0.5% (95% CI 0.4–0.7) in the first year and 1% (95% CI 0.8–1.4) in the fifth year. Graft failures were uncommon within the first year at 0.7% (95% CI 0.6–1.0), 1.2% (95% CI 0.9–1.5) by the second year and 2.5% (95% CI 2.1–3.0) by 5 years since entering the waitlist.

There were very few deaths at the end of the first year [1.4% (95% CI 1.2–1.7)], but this approximately doubled annually to 6.3% (95% CI 5.7–7.0) at 3 years. Thereafter, deaths increased by ≈3% each year and reached 12.8% (95% CI 11.9–13.8) by 5 years. Most deaths occurred while suspended or following a deceased donor transplant.

## DISCUSSION

Suspensions after entering the kidney waitlist were common, occurring in approximately one-third of our Australian national cohort overall. Prior suspensions had a compounding effect, making it about four times as likely to be suspended again and up to 50% more likely to return to the waitlist after suspension. Intersectional disadvantage was also evident, where only non-Indigenous males were more likely to return to the waitlist after suspension compared with their female counterparts. Prior suspensions led to reduced access to deceased donor transplantation by ≈30% and a 1-year longer wait when forecasting over a 5-year period. Being currently suspended had a considerable 12-fold increase in the risk of death while waiting.

To the best of our knowledge, no other studies have examined all patient transitions while on the kidney waitlist. Prior studies have instead focused on the return to the waitlist, permanent or first-year suspensions or outcomes after entering the kidney waitlist. Permanent suspensions occurred in 18% of the US kidney waitlisted population over a decade [12]. Similarly, suspensions in the first year of waitlisting occurred in 17% of the National Kidney Transplant Waiting List from the UK [13]. Our Australian study found a higher proportion of patients (37%) had experienced a suspension when considering all suspensions after entering the waitlist.

However, more favourable outcomes were seen in those ever suspended from our Australian population compared with those suspended in the first year from the UK population. Of those who were suspended in the first year in the UK, 45% were subsequently transplanted and 26% died while waiting. This compared with 66% transplanted and 12% who died while waiting in those who ever had a suspension in Australia. No other studies have examined returning to the waitlist, nor have they looked at how prior suspension episodes affect their risk of subsequent suspensions, returning to the waitlist, kidney transplantation and death while waiting.

Other factors associated with suspension in our cohort were similar to those reported for permanent suspensions in the US population [12]. There was an increased risk of permanent suspension in the USA among those of older age, male sex, African American ethnicity, with diabetes as cause of kidney failure and certain blood groups, as O and B were more likely and AB was less likely to be permanently suspended compared with A. Our study did find that blood group had the opposite effect on being suspended, where suspension was less likely in O and B and more likely in those with AB compared with A. This may reflect differences in the blood group distribution among the donor pool compared with the waitlisted population, which would impact who will be offered transplantation sooner. It is also important to note that waitlist dynamics will likely vary between and within countries. Countries or regions that rely more heavily on living donation or have shorter expected waiting times and more donors may have fewer patients being suspended because of the greater opportunity for transplantation to occur sooner.

Our novel finding is the disadvantage in return to the waitlist for those of Indigenous ethnicity and female sex, after adjusting for clinical factors. Similarly, African Americans and females were less likely to return to the waitlist in a US study [11]. However, this only considered the first instance of returning to the waitlist and not all suspensions. From other Australian studies, there is a known disadvantage for people of Indigenous ethnicity and female sex in being waitlisted and receiving a transplant, with substantial investment to redress [7, 10]. However, we found relisting after suspension was also a barrier; overall, people of Aboriginal and Torres Strait Islander ethnicity were 31% less likely to return to the waitlist after suspension and Indigenous males did not have the same advantage in returning to the waitlist as their non-Indigenous male counterparts. In addition, delays in accessing the waitlist results in higher priority once listed and should increase the likelihood of transplantation. Yet, Indigenous people do not have higher transplantation rates in the first year and have 60% lower likelihood of being transplanted after the first year of waitlisting. Our study similarly found Indigenous people were not overall more likely to receive a deceased donor transplant after entering the waitlist. Suspensions may play a role in this and offer an opportunity to improve health service delivery to get Indigenous people and females back onto the waitlist sooner, or to list pre-emptively, providing more possibilities for transplantation.

Our work has important implications for informing patients and providing more evidence to support informed discussions on the expected journey on the kidney waitlist in Australia. Before waitlisting, patients’ education is focused on what transplantation will mean for them and their health and the transplantation process once allocated a donor organ. Average waiting times are discussed, but they are less informed, if at all, about the potential for suspensions and any impact on the subsequent waitlisting outcome. Patients may find it helpful to know that suspensions occur commonly in one-third of those who have entered the waitlist, where most (>70%) would return to the waitlist. After 5 years on the waitlist, only 10% of patients would be suspended, 5% still waiting, 70% transplanted and <3% with graft failure. A reality that is rarely acknowledged or discussed with patients and their caregivers is that 13% of those waitlisted will die within 5 years. Suspensions may limit the window of opportunity for patients to be offered a transplant, extending the waitlisting time by 6–12 months and decreasing the likelihood of transplantation by ≈30%, but that does not mean patients won't receive a transplant. We found approximately half of those suspended would eventually be transplanted.

Our work also has important implications for further research. Organisation of the transplant centre's processes for managing the waitlist may partially impact the length and occurrence of suspensions. In the USA, low-performance centres, defined as those with the worst 1-year graft or patient survival, had a 60% higher risk of permanent suspension from the waitlist, despite having 10% lower mortality after suspension [23]. A focus on waitlist dynamics could provide an opportunity to design health interventions that improve health service organisation and facilitate a faster return to the waitlist for key disadvantaged groups. Our estimates of suspension, returning to the waitlist and death while waiting are also informative for economic health models. These could evaluate the cost of suspensions, or potential gains from redressing from a patient and health system perspective [24]. Our estimates could inform allocation algorithms, waitlist assessment and screening process design and conduct.

Our study is strengthened by the inclusion of the entire population who entered the kidney waitlist for a deceased donor transplant in Australia over more than a decade. Our findings reflect the Australian setting and may not be generalisable to other healthcare systems nor countries where transplantation processes and pathways differ (e.g. greater rates of living donor transplants). We also took a life course approach to consider the entire patient journey, providing annual estimates of all clinical states up to 5 years after entering the waitlist. However, our study did have limitations. We used data of waitlist status change that were not collected for research purposes. We were limited by waitlisting information that did not include the reason for suspension, expected time to relisting or whether the suspension was intended to be temporary or permanent, so we are unable to report these. Future data collection on the reason for suspension would be useful for further work to identify opportunities to minimise suspension occurrence and duration, particularly in certain patient groups. In addition, disentangling temporary and permanent suspensions may provide further insights into the associations between certain patient groups and likelihood of suspensions/returning to the waitlist.

In conclusion, the patient journey on the kidney waitlist was complex and not always a straightforward process. Suspensions occurred at least once in one in three Australian waitlisted patients. However, outcomes after suspension were generally encouraging; most would return to the waitlist and subsequently half would eventually be transplanted. Males were advantaged in returning to the waitlist sooner, particularly non-Indigenous males, and in receiving a deceased donor transplant. Our findings have provided evidence on the potential for suspensions and subsequent outcomes to support more informed discussions and shared decision making between patients and their clinicians. Future research should explore whether there are systematic biases in health service delivery for transplantation and whether health interventions could facilitate returning to the waitlist sooner.

## Supplementary Material

gfad253_Supplemental_File

## Data Availability

The data for this study were provided by ANZDATA. The patient-level data are not in the public domain due to restrictions set out in the data access agreement and our ethical oversight. Data may be available upon reasonable request to the corresponding author, subject to relevant approvals and permissions.
