# Supplementary material for: Journey to kidney transplantation: patient dynamics, suspensions, transplantation and deaths in the Australian kidney transplant waitlist
Source: Nephrol Dial Transplant. 2023 Nov 28;39(7):1138–49. doi: 10.1093/ndt/gfad253 (PMC11210985; doi:10.1093/ndt/gfad253)
Supplement: gfad253_Supplemental_File [file gfad253_supplemental_file.docx]

Supplementary methods

*Study design & setting*

ANZDATA does not include people with kidney failure who receive conservative (non-dialytic) care. Patient data is contributed in real-time and annually to ANZDATA from all dialysis and transplant units in Australia and New Zealand.

The former system to OrganMatch was the National Organ Matching Service (NOMS). Patient waitlisting information is limited and does not include reason for suspension, expected time to re-listing or whether suspension was expected to be temporary or permanent.

*Statistical analyses*

In the flexible parametric multi-state model, time was fitted as cohort discrete time state transition model (cDTSTM) with a time inhomogeneous Markov process, where t was measured from the initial state of entering the waitlist until the first transplant 1. The cause-specific hazard ratios with values greater than one indicated the transition was more likely to occur and values less than one indicated the transition was less likely to occur, compared with remaining in the same state.

Likelihood ratio tests were used to test the significance of the covariates and interaction terms in the flexible parametric multi-state models. Wald tests were used to test the significance in the Cox univariable and multivariable models.

The ‘strmst’ package fits a flexible parametric survival model using restricted cubic spline functions through the dependency package ‘stpm2’ in Stata 2. This is akin to our above flexible parametric multi-state models, however it is only used for clinical endpoints as it does not consider multi-states and takes a spline-based approach. The advantages of this approach compared to the Cox model are smoother predictions over time and modelling of more complex time-dependent effects 2. We used two knots for the baseline spline function and treated the time-dependent variable as a linear effect of log time.

*References*

1. Briggs AH, Claxton K, Sculpher MJ. *Decision modelling for health economic evaluation.* Oxford: Oxford University Press; 2006.

2. Lambert PC, Royston P. Further development of flexible parametric models for survival analysis. *Stata J.* 2009;9(2):265-290.

Supplementary Figure S1. Distribution of time since waitlisting and time since dialysis until first transplant or 31^st^ Dec 2019 for the entire study population, by blood group.

Supplementary Table S1. Summary of 3-monthly rates (per 100,000 person-years) of being suspended, death while active on waitlist and death while suspended.

| **Transition** | **Events** | **Rate per 100,000 person-years (95% CI)** | |
| --- | --- | --- | --- |
| Rate of being suspended | 4797 | 50479.0 | (49071.0-51928.0) |
| Overall |  | 12619.8 | (12267.8-12982.0) |
| 1st year |  | 12727.3 | (12236.3-13238.0) |
| >1st year |  | 12506.5 | (12007.3-13026.5) |
| Rate of death while active on waitlist | 31 | 326.2 | (229.4-463.9) |
| Overall |  | 81.6 | (57.4-116.0) |
| 1st year |  | 61.5 | (35.0-108.4) |
| >1st year |  | 102.7 | (65.5-160.9) |
| Rate of death while suspended | 350 | 7438.0 | (6698.2-8259.5) |
| Overall |  | 1859.5 | (1674.6-2064.9) |
| 1st year |  | 841.3 | (559.1-1266.0) |
| >1st year |  | 2032.5 | (1823.7-2265.2) |

Supplementary Table S2. Univariable flexible parametric multi-state model estimates of hazard ratios associated with transitioning from active to suspended and from suspended to active. P-value from likelihood ratio test.

|  | **Active to suspended** | | | **Suspended to active** | | |
| --- | --- | --- | --- | --- | --- | --- |
|  | **HR** | **(95% CI)** | **p-value** | **HR** | **(95% CI)** | **p-value** |
| **Sex** |  |  | 0.390 |  |  | 0.984 |
| Female | 1.00 |  |  | 1.00 |  |  |
| Male | 1.03 | (0.97, 1.09) |  | 1.00 | (0.94, 1.07) |  |
| **Age at waitlist entry (years)** |  |  | **<0.001** |  |  | **<0.001** |
| ≤29 | 1.26 | (1.14, 1.39) |  | 1.11 | (0.99, 1.24) |  |
| 30-49 | 1.00 |  |  | 1.00 |  |  |
| 50-64 | 1.14 | (1.07, 1.22) |  | 0.82 | (0.76, 0.88) |  |
| ≥65 | 1.27 | (1.15, 1.39) |  | 0.56 | (0.50, 0.63) |  |
| **Year of KRT** |  |  | **<0.001** |  |  | **<0.001** |
| ≤2007 | 1.00 |  |  | 1.00 |  |  |
| 2008-11 | 1.09 | (1.00, 1.18) |  | 1.46 | (1.33, 1.60) |  |
| 2012-15 | 1.28 | (1.18, 1.38) |  | 2.25 | (2.06, 2.46) |  |
| 2016-19 | 1.27 | (1.16, 1.39) |  | 2.88 | (2.59, 3.19) |  |
| **Ethnicity*** |  |  | **<0.001** |  |  | **<0.001** |
| Australian & New Zealander | 1.00 |  |  | 1.00 |  |  |
| Aboriginal & Torres Strait Islander | 1.71 | (1.52, 1.92) |  | 0.71 | (0.62, 0.81) |  |
| Maori & Pacific Islander | 1.12 | (0.98, 1.29) |  | 0.81 | (0.68, 0.95) |  |
| Asian | 0.96 | (0.89, 1.04) |  | 1.37 | (1.27, 1.49) |  |
| Other | 0.94 | (0.85, 1.03) |  | 1.65 | (1.48, 1.83) |  |
| **Blood group** |  |  | **0.003** |  |  | **<0.001** |
| A | 1.00 |  |  | 1.00 |  |  |
| AB | 1.21 | (0.99, 1.49) |  | 0.93 | (0.74, 1.16) |  |
| B | 0.93 | (0.85, 1.01) |  | 1.40 | (1.28, 1.55) |  |
| O | 0.91 | (0.85, 0.97) |  | 1.09 | (1.01, 1.17) |  |
| **Prior suspensions** |  |  | **<0.001** |  |  | **<0.001** |
| 0 | 1.00 |  |  | 1.00 |  |  |
| 1 | 3.25 | (3.02, 3.48) |  | 1.12 | (1.04, 1.22) |  |
| 2 | 4.00 | (3.68, 4.35) |  | 1.49 | (1.36, 1.64) |  |
| **Comorbidity Count** |  |  | **<0.001** |  |  | **<0.001** |
| 0 | 1.00 |  |  | 1.00 |  |  |
| 1 | 1.31 | (1.22, 1.40) |  | 0.72 | (0.67, 0.77) |  |
| 2 | 1.61 | (1.46, 1.77) |  | 0.52 | (0.46, 0.58) |  |
| 3+ | 1.64 | (1.43, 1.89) |  | 0.46 | (0.39, 0.55) |  |
| **Cause of Kidney Failure** |  |  | **<0.001** |  |  | **<0.001** |
| Diabetes | 1.64 | (1.52, 1.77) |  | 0.68 | (0.63, 0.75) |  |
| Hypertension/renal artery disease | 1.13 | (1.02, 1.26) |  | 0.70 | (0.62, 0.79) |  |
| Glomerulonephritis/IgA nephropathy | 1.00 |  |  | 1.00 |  |  |
| Polycystic kidney disease | 0.97 | (0.89, 1.06) |  | 1.25 | (1.13, 1.38) |  |
| Other | 1.19 | (1.10, 1.29) |  | 1.01 | (0.93, 1.10) |  |
| **Australia state/territory** |  |  | **<0.001** |  |  | **<0.001** |
| One | 1.00 |  |  | 1.00 |  |  |
| Two | 1.54 | (1.32, 1.80) |  | 0.99 | (0.83, 1.17) |  |
| Three | 3.26 | (2.78, 3.81) |  | 1.85 | (1.56, 2.20) |  |
| Four | 1.29 | (1.18, 1.41) |  | 1.05 | (0.94, 1.16) |  |
| Five | 1.10 | (0.96, 1.27) |  | 1.71 | (1.47, 1.99) |  |
| Six | 1.57 | (1.27, 1.94) |  | 1.18 | (0.94, 1.50) |  |
| Seven | 1.15 | (1.08, 1.24) |  | 1.47 | (1.36, 1.59) |  |
| Eight | 1.60 | (1.42, 1.81) |  | 1.33 | (1.16, 1.53) |  |
| **Interaction terms** |  |  |  |  |  |  |
| Sex and ethnicity |  |  | 0.371 |  |  | **<0.001** |

*Categorized based on the Australian Standard Classification of Cultural and Ethnic Groups 2016.

Supplementary Table S3. Multivariable flexible parametric multi-state model estimates of hazard ratios associated with transitioning from active to suspended and from suspended to active. P-value from likelihood ratio test.

|  | **Waitlist to suspended** | | | **Suspended to waitlist** | | |
| --- | --- | --- | --- | --- | --- | --- |
|  | **HR** | **(95% CI)** | **p-value** | **HR** | **(95% CI)** | **p-value** |
| **Sex** |  |  | 0.070 |  |  | **0.004** |
| Female | 1.00 |  |  | 1.00 |  |  |
| Male | 1.03 | (0.97, 1.09) |  | 1.10 | (1.03, 1.18) |  |
| **Age at waitlist entry (years)** |  |  | **0.006** |  |  | **<0.001** |
| ≤29 | 1.21 | (1.09, 1.34) |  | 0.98 | (0.88, 1.10) |  |
| 30-49 | 1.00 |  |  | 1.00 |  |  |
| 50-64 | 1.09 | (1.01, 1.16) |  | 0.93 | (0.86, 1.00) |  |
| ≥65 | 1.21 | (1.10, 1.34) |  | 0.60 | (0.54, 0.68) |  |
| **Year of KRT** |  |  | **<0.001** |  |  | **<0.001** |
| ≤2007 | 1.00 |  |  | 1.00 |  |  |
| 2008-11 | 1.08 | (0.99, 1.17) |  | 1.42 | (1.30, 1.56) |  |
| 2012-15 | 1.26 | (1.16, 1.37) |  | 2.21 | (2.02, 2.42) |  |
| 2016-19 | 1.46 | (1.33, 1.60) |  | 2.70 | (2.42, 3.02) |  |
| **Ethnicity*** |  |  | 0.045 |  |  | **<0.001** |
| Australian & New Zealander | 1.00 |  |  | 1.00 |  |  |
| Aboriginal & Torres Strait Islander | 1.07 | (0.93, 1.23) |  | 0.69 | (0.59, 0.81) |  |
| Māori & Pacific Islander | 0.88 | (0.76, 1.01) |  | 0.89 | (0.75, 1.06) |  |
| Asian | 0.92 | (0.85, 1.00) |  | 1.18 | (1.08, 1.29) |  |
| Other | 0.95 | (0.86, 1.05) |  | 1.20 | (1.08, 1.35) |  |
| **Blood group** |  |  | **<0.001** |  |  | **<0.001** |
| A | 1.00 |  |  | 1.00 |  |  |
| AB | 1.31 | (1.07, 1.62) |  | 0.82 | (0.65, 1.03) |  |
| B | 0.82 | (0.75, 0.89) |  | 1.23 | (1.11, 1.36) |  |
| O | 0.81 | (0.76, 0.87) |  | 1.02 | (0.94, 1.10) |  |
| **Prior suspensions** |  |  | **<0.001** |  |  | **<0.001** |
| 0 | 1.00 |  |  | 1.00 |  |  |
| 1 | 3.37 | (3.13, 3.62) |  | 1.21 | (1.12, 1.31) |  |
| 2+ | 4.18 | (3.83, 4.57) |  | 1.50 | (1.36, 1.65) |  |
| **Comorbidity Count** |  |  | **<0.001** |  |  | **<0.001** |
| 0 | 1.00 |  |  | 1.00 |  |  |
| 1 | 1.22 | (1.13, 1.30) |  | 0.79 | (0.73, 0.86) |  |
| 2 | 1.37 | (1.23, 1.51) |  | 0.67 | (0.59, 0.75) |  |
| 3+ | 1.58 | (1.36, 1.82) |  | 0.61 | (0.51, 0.72) |  |
| **Cause of Kidney Failure** |  |  | **<0.001** |  |  | **<0.001** |
| Diabetes | 1.32 | (1.21, 1.44) |  | 0.67 | (0.61, 0.73) |  |
| Hypertension/renal artery disease | 1.07 | (0.96, 1.20) |  | 0.80 | (0.70, 0.90) |  |
| Glomerulonephritis/IgA nephropathy | 1.00 |  |  | 1.00 |  |  |
| Polycystic kidney disease | 0.95 | (0.86, 1.04) |  | 1.17 | (1.06, 1.30) |  |
| Other | 1.08 | (0.99, 1.17) |  | 0.96 | (0.88, 1.06) |  |
| **Australia state/territory** |  |  | **<0.001** |  |  | **<0.001** |
| One | 1.00 |  |  | 1.00 |  |  |
| Two | 1.30 | (1.11, 1.53) |  | 1.08 | (0.91, 1.29) |  |
| Three | 2.04 | (1.69, 2.45) |  | 2.00 | (1.65, 2.44) |  |
| Four | 1.42 | (1.29, 1.55) |  | 0.89 | (0.80, 0.99) |  |
| Five | 1.15 | (0.99, 1.32) |  | 1.52 | (1.30, 1.78) |  |
| Six | 1.49 | (1.19, 1.85) |  | 1.19 | (0.93, 1.51) |  |
| Seven | 1.18 | (1.10, 1.26) |  | 1.24 | (1.14, 1.34) |  |
| Eight | 1.80 | (1.59, 2.04) |  | 1.46 | (1.27, 1.69) |  |
| **Interaction terms** |  |  |  |  |  |  |
| Sex and ethnicity |  |  | 0.256 |  |  | **<0.001** |

*Categorized based on the Australian Standard Classification of Cultural and Ethnic Groups 2016.

Supplementary Table S4. Multivariate flexible parametric multi-state model estimates of hazard ratios for interaction term associated with transitioning from suspended to active. P value from Wald test.

| **Suspended to waitlist** | **Univariate** | | | **Multivariate** | | |
| --- | --- | --- | --- | --- | --- | --- |
|  | **HR** | **(95% CI)** | **p-value** | **HR** | **(95% CI)** | **p-value** |
| *Sex and ethnicity* | | | | | | |
| Australian & New Zealander |  |  |  |  |  |  |
| Female | 1.00 |  |  | 1.00 |  |  |
| Male | 1.00 | (0.92, 1.09) | 0.930 | 1.13 | (1.04, 1.23) | **0.006** |
| Aboriginal & Torres Strait Islander |  |  |  |  |  |  |
| Female | 1.00 |  |  | 1.00 |  |  |
| Male | 1.03 | (0.79, 1.34) | 0.818 | 1.27 | (0.97, 1.65) | 0.083 |
| Māori & Pacific Islander |  |  |  |  |  |  |
| Female | 1.00 |  |  | 1.00 |  |  |
| Male | 1.11 | (0.80, 1.52) | 0.540 | 1.33 | (0.96, 1.84) | 0.082 |
| Asian |  |  |  |  |  |  |
| Female | 1.00 |  |  | 1.00 |  |  |
| Male | 1.06 | (0.92, 1.22) | 0.424 | 1.23 | (1.06, 1.42) | **0.006** |
| Other |  |  |  |  |  |  |
| Female | 1.00 |  |  | 1.00 |  |  |
| Male | 0.62 | (0.51, 0.76) | **<0.001** | 0.64 | (0.52, 0.78) | **<0.001** |

Supplementary Table S5. Univariable cause-specific Cox model for factors associated from: waitlist entry to transplant (deceased donor), and waitlist entry to death before transplant.

|  | **Waitlist entry to deceased donor transplant** | | | **Waitlist entry to death before transplant** | | |
| --- | --- | --- | --- | --- | --- | --- |
|  | **HR** | **(95% CI)** | **p-value** | **HR** | **(95% CI)** | **p-value** |
| **Sex** |  |  | **<0.001** |  |  | **0.007** |
| Female | 1.00 |  |  | 1.00 |  |  |
| Male | 1.10 | (1.04-1.16) |  | 1.34 | (1.08-1.65) |  |
| **Age at waitlist entry (years)** |  |  | **<0.001** |  |  | **<0.001** |
| ≤29 | 1.20 | (1.09-1.31) | <0.001 | 0.59 | (0.31-1.10) | 0.097 |
| 30-49 | 1.00 |  |  | 1.00 |  |  |
| 50-64 | 1.09 | (1.03-1.16) | 0.003 | 2.10 | (1.61-2.72) | <0.001 |
| ≥65 | 1.19 | (1.09-1.29) | <0.001 | 2.99 | (2.21-4.06) | <0.001 |
| **Year of KRT** |  |  | **<0.001** |  |  | 0.319 |
| ≤2007 | 1.00 |  |  | 1.00 |  |  |
| 2008-11 | 1.17 | (1.09-1.26) | <0.001 | 0.97 | (0.76-1.24) | 0.792 |
| 2012-15 | 1.52 | (1.42-1.64) | <0.001 | 1.25 | (0.94-1.67) | 0.126 |
| 2016-19 | 1.41 | (1.29-1.53) | <0.001 | 0.97 | (0.64-1.48) | 0.888 |
| **Ethnicity*** |  |  | **<0.001** |  |  | **0.019** |
| Australian & New Zealander | 1.00 |  |  | 1.00 |  |  |
| Aboriginal & Torres Strait Islander | 0.96 | (0.86-1.08) | 0.497 | 1.23 | (0.85-1.77) | 0.266 |
| Maori & Pacific Islander | 0.78 | (0.68-0.90) | <0.001 | 1.06 | (0.67-1.67) | 0.807 |
| Asian | 0.74 | (0.69-0.80) | <0.001 | 0.77 | (0.58-1.03) | 0.073 |
| Other | 1.03 | (0.95-1.11) | 0.501 | 0.48 | (0.28-0.83) | 0.008 |
| **Blood group** |  |  | **<0.001** |  |  | 0.612 |
| A | 1.00 |  |  | 1.00 |  |  |
| AB | 1.80 | (1.60-2.02) | <0.001 | 0.73 | (0.32-1.66) | 0.453 |
| B | 0.58 | (0.54-0.63) | <0.001 | 0.87 | (0.64-1.20) | 0.411 |
| O | 0.62 | (0.58-0.65) | <0.001 | 0.87 | (0.69-1.09) | 0.234 |
| **Clinical state at death** |  |  |  |  |  | **<0.001** |
| Active |  |  |  | 1.00 |  |  |
| Suspended |  |  |  | 15.10 | (10.12-22.53) |  |
| **Prior suspensions** |  |  | **<0.001** |  |  | 0.098 |
| 0 | 1.00 |  |  | 1.00 |  |  |
| 1 | 0.64 | (0.57-0.71) | <0.001 | 0.88 | (0.68-1.15) | 0.355 |
| 2 | 0.75 | (0.65-0.86) | <0.001 | 0.69 | (0.48-0.98) | 0.037 |
| **Comorbidity Count** |  |  | 0.313 |  |  | **<0.001** |
| 0 | 1.00 |  |  | 1.00 |  |  |
| 1 | 1.03 | (0.97-1.10) | 0.280 | 2.41 | (1.89-3.08) | <0.001 |
| 2 | 0.96 | (0.87-1.05) | 0.346 | 3.36 | (2.53-4.47) | <0.001 |
| 3+ | 0.94 | (0.82-1.07) | 0.349 | 4.00 | (2.84-5.63) | <0.001 |
| **Cause of Kidney Failure** |  |  | **<0.001** |  |  | **<0.001** |
| Diabetes | 0.91 | (0.85-0.98) | 0.010 | 3.32 | (2.57-4.31) | <0.001 |
| Hypertension/renal artery disease | 0.88 | (0.80-0.97) | 0.009 | 1.48 | (1.01-2.16) | 0.043 |
| Glomerulonephritis/IgA nephropathy | 1.00 |  |  | 1.00 |  |  |
| Polycystic kidney disease | 1.08 | (1.00-1.16) | 0.056 | 0.88 | (0.58-1.34) | 0.547 |
| Other | 1.14 | (1.07-1.22) | <0.001 | 1.36 | (0.99-1.88) | 0.056 |
| **Australia state/territory** |  |  | **<0.001** |  |  | 0.057 |
| One | 1 |  |  | 1.00 |  |  |
| Two | 0.81 | (0.66-1.00) | 0.054 | 1.02 | (0.64-1.62) | 0.938 |
| Three | 1.61 | (1.33-1.95) | <0.001 | 0.36 | (0.09-1.46) | 0.154 |
| Four | 2.36 | (2.19-2.54) | <0.001 | 0.59 | (0.40-0.87) | 0.007 |
| Five | 2.84 | (2.58-3.13) | <0.001 | 0.89 | (0.48-1.63) | 0.699 |
| Six | 1.75 | (1.44-2.12) | <0.001 | 1.12 | (0.53-2.39) | 0.766 |
| Seven | 1.54 | (1.44-1.65) | <0.001 | 0.93 | (0.72-1.20) | 0.582 |
| Eight | 2.83 | (2.57-3.11) | <0.001 | 1.41 | (0.94-2.12) | 0.094 |
| **Interaction terms** |  |  |  |  |  |  |
| Sex and ethnicity |  |  | 0.208 |  |  | 0.751 |

*Categorized based on the Australian Standard Classification of Cultural and Ethnic Groups 2016.

Supplementary Table S6. Multivariable cause-specific Cox model for factors associated from: waitlist entry to transplant (deceased donor), and waitlist entry to death before transplant.

|  | **Waitlist entry to deceased donor transplant** | | | **Waitlist entry to death before transplant** | | |
| --- | --- | --- | --- | --- | --- | --- |
|  | **HR** | **(95% CI)** | **p-value** | **HR** | **(95% CI)** | **p-value** |
| **Sex** |  |  | **<0.001** |  |  | 0.775 |
| Female | 1.00 |  |  | 1.00 |  |  |
| Male | 1.11 | (1.05-1.17) |  | 1.03 | (0.83-1.29) |  |
| **Age at waitlist entry (years)** |  |  | **<0.001** |  |  | **<0.001** |
| ≤29 | 1.08 | (0.98-1.18) | 0.125 | 0.58 | (0.30-1.10) | 0.095 |
| 30-49 | 1.00 |  |  | 1.00 |  |  |
| 50-64 | 1.10 | (1.03-1.17) | 0.002 | 1.46 | (1.11-1.92) | 0.006 |
| ≥65 | 1.23 | (1.13-1.34) | <0.001 | 1.92 | (1.39-2.66) | <0.001 |
| **Year of KRT** |  |  | **<0.001** |  |  | 0.431 |
| ≤2007 | 1.00 |  |  | 1.00 |  |  |
| 2008-11 | 1.19 | (1.11-1.29) | <0.001 | 0.98 | (0.76-1.26) | 0.873 |
| 2012-15 | 1.59 | (1.48-1.72) | <0.001 | 1.24 | (0.91-1.67) | 0.168 |
| 2016-19 | 1.40 | (1.28-1.52) | <0.001 | 1.16 | (0.75-1.82) | 0.505 |
| **Ethnicity*** |  |  | **<0.001** |  |  | 0.195 |
| Australian & New Zealander | 1.00 |  |  | 1.00 |  |  |
| Aboriginal & Torres Strait Islander | 0.91 | (0.80-1.03) | 0.135 | 0.92 | (0.62-1.37) | 0.672 |
| Māori & Pacific Islander | 0.92 | (0.80-1.06) | 0.253 | 0.86 | (0.53-1.37) | 0.521 |
| Asian | 0.87 | (0.80-0.94) | <0.001 | 0.78 | (0.57-1.06) | 0.113 |
| Other | 1.07 | (0.99-1.17) | 0.089 | 0.56 | (0.32-0.98) | 0.041 |
| **Blood group** |  |  | **<0.001** |  |  | 0.405 |
| A | 1.00 |  |  | 1.00 |  |  |
| AB | 1.72 | (1.53-1.93) | <0.001 | 0.70 | (0.30-1.64) | 0.416 |
| B | 0.60 | (0.56-0.66) | <0.001 | 1.27 | (0.91-1.77) | 0.162 |
| O | 0.57 | (0.54-0.60) | <0.001 | 1.07 | (0.85-1.35) | 0.553 |
| **Clinical state at death** |  |  |  |  |  | **<0.001** |
| Active |  |  |  | 1.00 |  |  |
| Suspended |  |  |  | 12.12 | (8.04-18.30) |  |
| **Prior suspensions** |  |  | **<0.001** |  |  | **0.017** |
| 0 | 1.00 |  |  | 1.00 |  |  |
| 1 | 0.65 | (0.58-0.72) | <0.001 | 0.69 | (0.52-0.91) | 0.008 |
| 2+ | 0.71 | (0.62-0.82) | <0.001 | 0.74 | (0.51-1.08) | 0.115 |
| **Comorbidity Count** |  |  | 0.127 |  |  | **<0.001** |
| 0 | 1.00 |  |  | 1.00 |  |  |
| 1 | 1.06 | (1.00-1.13) | 0.063 | 1.61 | (1.24-2.08) | <0.001 |
| 2 | 1.00 | (0.91-1.10) | 0.942 | 1.61 | (1.18-2.20) | 0.003 |
| 3+ | 1.13 | (0.98-1.29) | 0.090 | 1.86 | (1.28-2.71) | 0.001 |
| **Cause of Kidney Failure** |  |  | **<0.001** |  |  | **<0.001** |
| Diabetes | 0.87 | (0.80-0.94) | <0.001 | 1.93 | (1.45-2.57) | <0.001 |
| Hypertension/renal artery disease | 0.86 | (0.78-0.95) | 0.003 | 1.04 | (0.71-1.55) | 0.827 |
| Glomerulonephritis/IgA nephropathy | 1.00 |  |  | 1.00 |  |  |
| Polycystic kidney disease | 1.01 | (0.93-1.09) | 0.899 | 0.89 | (0.58-1.36) | 0.582 |
| Other | 1.04 | (0.98-1.12) | 0.214 | 1.28 | (0.92-1.77) | 0.149 |
| **Australia state/territory** |  |  | **<0.001** |  |  | **0.015** |
| One | 1 |  |  | 1 |  |  |
| Two | 0.85 | (0.69-1.04) | 0.115 | 0.79 | (0.48-1.28) | 0.335 |
| Three | 2.03 | (1.65-2.50) | <0.001 | 0.24 | (0.06-1.01) | 0.051 |
| Four | 2.42 | (2.25-2.61) | <0.001 | 0.55 | (0.37-0.82) | 0.003 |
| Five | 2.88 | (2.61-3.18) | <0.001 | 0.69 | (0.37-1.29) | 0.242 |
| Six | 1.66 | (1.37-2.01) | <0.001 | 0.72 | (0.33-1.56) | 0.402 |
| Seven | 1.55 | (1.45-1.66) | <0.001 | 0.82 | (0.63-1.06) | 0.123 |
| Eight | 3.35 | (3.04-3.69) | <0.001 | 1.28 | (0.84-1.95) | 0.246 |
| **Interaction terms** |  |  |  |  |  |  |
| Sex and ethnicity |  |  | 0.054 |  |  | 0.855 |

*Categorized based on the Australian Standard Classification of Cultural and Ethnic Groups 2016.
